# Embryonic and postnatal telomere length decrease with ovulation order within clutches

**DOI:** 10.1038/srep25915

**Published:** 2016-05-13

**Authors:** José C. Noguera, Neil B. Metcalfe, Sophie Reichert, Pat Monaghan

**Affiliations:** 1 Institute of Biodiversity Animal Health & Comparative Medicine, College of Medical, Veterinary & Life Sciences, Graham Kerr Building, University of Glasgow, Glasgow G12 8QQ, UK

## Abstract

Telomere length (TL) in early life has been found to be predictive of subsequent lifespan. Factors such as parental TL, parental age and environmental conditions during development have been shown to contribute to the observed variation in TL among individuals. One factor that has not hitherto been considered is ovulation order, although it is well established that the last hatched/born offspring in a brood or litter often show relatively poor subsequent performance. We examined the within- and across-clutch effect of ovulation order on TL in embryos of zebra finches experiencing the same controlled incubation conditions (N = 151), and tested whether any such ovulation order effects remained detectable in adults (N = 122). Irrespective of clutch and egg size, TL in early-stage embryos (72 h incubation) markedly decreased with within-clutch ovulation order; the difference in TL of first and last-laid embryos was equivalent to the average within-individual telomere loss over the entire period of nestling and juvenile life. This ovulation-order effect occurred only within but not across clutches, and was still evident in adults. Given that TL in early life predicts lifespan, our results suggest that parental effects on telomere length could contribute to the known poor performance of later-ovulated family members.

Telomeres are the evolutionarily conserved caps found at the ends of chromosomes that serve both to identify the true chromosome ends, and protect the coding sequences from the erosion that occurs during DNA replication (reviewed by^1^). In the absence of restoration, telomeres consequently shorten with each cell division but also as a result of oxidative stress^2^, and, when critically short, they cease to function effectively, triggering cell replicative senescence. Such senescent cells then either remain but with an altered secretory profile, or undergo apoptosis^3^. Telomere shortening is therefore associated with ageing. Variation in the rate of telomere shortening has been linked to variation organismal-level patterns of disease and senescence^4^,^5^, with individuals having shorter telomeres or higher loss rates often showing increased risk of diseases (reviewed by^6^) and reduced survival^7^,^8^.

Recently, a longitudinal study has revealed that measures of telomere length (TL) in early life can be a good predictor of longevity^9^. Consequently, factors that influence TL during early development are likely to be particularly important since they might affect later lifelong performance. Evidence is accumulating that postnatal exposure to different kinds of environmental stressors such as environmental pollution^10^, social and psychological stress^8^,^11^ or nutritional deficiencies^12^ can all accelerate the rate of telomere loss, possibly related to the fact that telomere shortening is greater after increased exposure to stress hormones (see i.e.^13^,^14^). Prenatal factors can also be important; these include paternal age at conception, which has been shown to influence offspring TL^15^,^16^, as well as the concentration of stress hormones in the egg^13^. However, one factor that has not hitherto been investigated is ovulation order.

In many vertebrate species, including humans, offspring are sequentially produced by consecutive ovulation. While this occurs across reproductive events in iteroparous species, it also occurs within a single reproductive event in species that produce multiple oocytes in each event (i.e. most birds, reptiles, egg-laying mammals and some amphibians and insects). In some taxa (e.g. birds) there is a clear laying order in the eggs produced within a clutch, since a single egg is ovulated and laid every 1**–**2 days^17^. However, egg production is costly^18^ and mothers can also strategically influence egg traits^19^. As a result, egg size within broods can vary^20^, as can egg composition i.e. antioxidant and hormonal content^19^,^21^. Such within-clutch variation in egg size and composition has consequences for the offspring, with generally a reduced size, viability and performance of the last produced embryos/chicks^22^,^23^,^24^. However, offspring from the last laid/hatched eggs can have impaired performance and survival prospects even when parental nutritional resources do not appear to be limiting and the last-laid eggs in a brood are no smaller that the first laid^25^,^26^,^27^,^28^. Given the relationship between TL and individual performance and lifespan, we therefore hypothesised that the observed reduction in viability and performance of the last offspring in a brood might be related to trends in TL with ovulation/laying order.

We investigated the effect of ovulation order on TL in the zebra finch *Taeniopygia guttata* both within and across clutches. In this species, females lay an average of four eggs per clutch (typical range 2–6) at a rate of one egg per day^29^. While egg size often increases with laying order in this species, the maternal allocation of antioxidants decreases^30^ and the last hatched chick often shows reduced performance i.e. lower growth and survival rate^31^. Moreover, early postnatal TL in this species is variable^9^,^12^, with individuals with shorter telomeres having a reduced lifespan^9^. We therefore examined the relationship between laying order and TL in early embryos. By allowing our study females to lay two clutches sequentially, we also examined whether any decline in telomere length in embryos from a first clutch continued into the next clutch or not. In a separate group of birds that were allowed to hatch and grow into adults, we examined their TL at different post-hatching ages to see if any effects of laying order detected very early in pre-natal development persisted into later life stages.

## Results

### Egg mass

**Figure 1:**
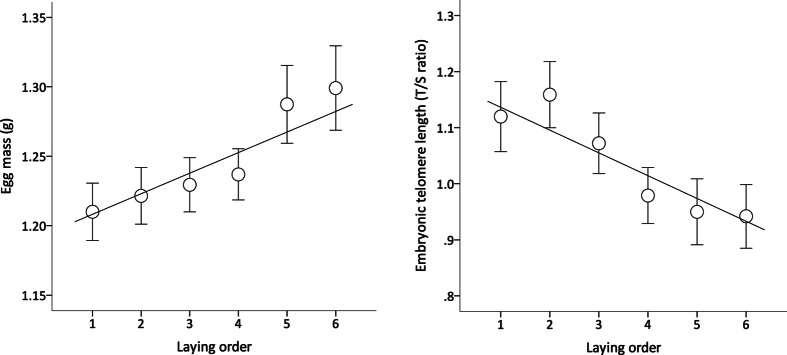
Relationship between the position of a zebra finch egg within a clutch (i.e. its laying order) and its mass and TL after 72 h of incubation (N = 151). (**a**) Egg mass (mean ± SE) increased with laying order whereas (**b**) TL in early-stage embryos (mean ± SE) decreased with position of the egg in the laying order within the clutch, irrespective of clutch size. Solid lines show linear regressions (see text for statistical analysis and Figure S1 for further details).

Egg mass slightly increased with laying order (Table 1; Fig. 1a), an effect that was independent of clutch size (clutch size x laying order: F_1,124.90_ = 0.026, p = 0.873) and similar in both clutches produced by the study females (effect of clutch number: estimate = 0.023, F_1,135.43_ = 3.478, p = 0.064; clutch number x laying order: estimate = 0.012, F_1,121.85_ = 2.829, p = 0.095). Egg mass also varied with the sex of the embryo (Table 1); the egg size of male embryos was, on average, 1.6% heavier than for female embryos. The mother’s body mass at the time of pairing (estimate = 0.018, F_1,47.96_ = 2.921, p = 0.094), clutch size (estimate = 0.021, F_1,62.091_ = 3.587, p = 0.063) and the remaining two-way interactions had no effect on egg mass (all p>0.587).

### Pre and postnatal telomere length

**Figure 2:**
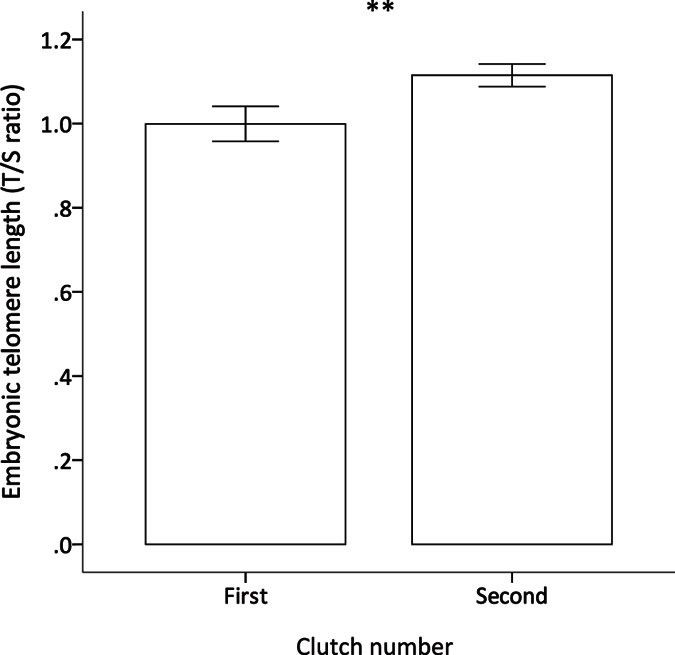
Early-stage embryo TL in successive clutches (N = 151). On average, embryos from the first clutches of female zebra finches had shorter TL (mean ± se) than those from the second clutches (see Figure S2 for further details).

Our exploratory analyses showed that TL in early-stage embryos decreased with position of the egg in the laying order within the clutch (F_1,137.75_ = 10.893, p < 0.001; Table 1; Fig. 1b) irrespective of clutch size (clutch size x laying order: estimate = −0.029, F_1,138.71_ = 3.004, p = 0.085). On average, embryos from the first clutches of each female had 11.1% shorter TL than those from the second clutches (Table 1; Fig. 2), but the effect on TL of laying order within a clutch was the same in both clutches (clutch number x laying order: F_1,134.254_ = 0.781, p = 0.378). The mother’s body mass (F_1,17.54_ = 1.093, p = 0.310), sex of the embryo (F_1,136.74_ = 0.015, p = 0.902), clutch size (F_1,59.48_ = 0.043, p = 0.836), egg mass (F_1,65.67_ = 0.050, p = 0.823) and the remaining two-way interactions (all p > 0.341) had no effect on embryonic TL.

**Figure 3:**
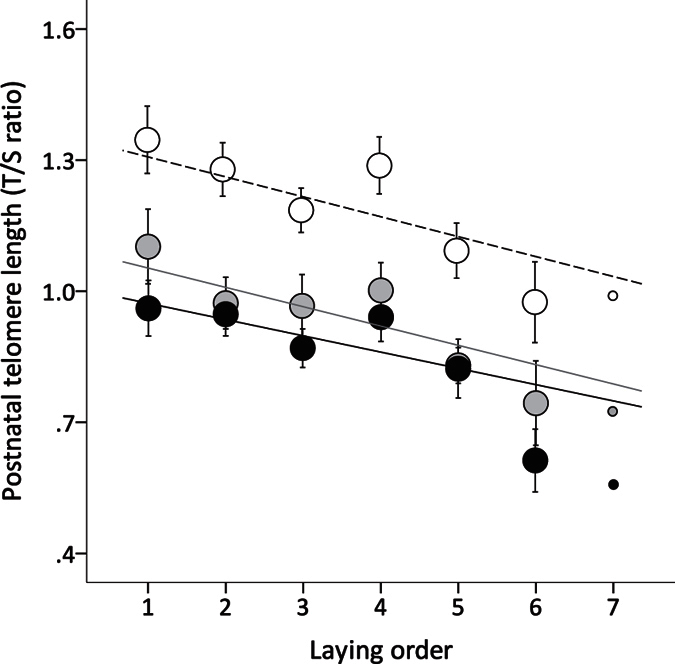
Laying order and postnatal telomere dynamics (N = 122). Relationship between the original position of a zebra finch within a clutch (i.e. its laying order) and its telomere length (mean ± SE) at 20 (white dots and dashed line), 40 (grey dots and solid grey line) and 90 days of age (black dots and solid black lines). Lines show the adjusted regression lines. There was a significant effect of laying order on telomere length which was not affected by age (see text for statistical analysis and Figure S3 for further details).

The samples taken from the birds that developed into adults showed that TL shortened with age (F_2,254.10_ = 41.911, p < 0.001); despite this effect, and after controlling for all factors previously shown to have a significant influence on postnatal TL (see Noguera *et al*.^12^ and Table S1 in Supplementary Information) there remained a consistent negative relationship between a bird’s original position within its clutch (laying order) and its TL up to at least early adulthood (90 days) (laying order: F_1,326.61_ = 18.726, p < 0.001; age x laying order: F_2,232.13_ = 0.364, p = 0.695; Fig. 3). Neither clutch size (F_1,321.62_ = 1.323, p = 0.251) nor any three and two-way interactions among age, laying order and clutch size were statistically significant (all p > 0.367).

## Discussion

Understanding the physiological mechanisms involved in early-life effects on health and lifespan is an increasing focus of many different biological and biomedical studies. In this study we have provided, for the first time, evidence of a short- and long-term effect of within-clutch ovulation order on prenatal telomere length and postnatal telomere dynamics. We found that TL in early-stage zebra finch embryos decreased with laying order within a clutch, and that this effect persisted well into postnatal life: nestlings hatched from the last-laid eggs of a clutch had consistently shorter TL throughout their development and through to at least early adulthood. The scale of this effect was substantial: following the method used by Heidinger *et al*.^9^ in the same species, we can calculate a rough estimate of postnatal telomere loss in terms of base pairs. On average, postnatal TL in last-laid eggs (in an average clutch size of 4 eggs) were 814 bp shorter that the TL of the first-laid eggs in the same clutch. This difference is similar in magnitude to the loss observed during postnatal development through to sexual maturity (TL reduction of 835 bp on average between 20–90 days of age), and is equivalent to approximately 60% of the total loss reported for the first year of life in this and other bird species approx., 1300bp; see^9^ and references therein. Since reductions in TL of this magnitude are linked to organismal level outcomes^9^,^32^,^33^, including significant reductions in longevity^9^,^34^, our results suggest that shortened telomeres from early in embryonic development may be an important, and so far unreported, physiological mechanism contributing to the negative influence of laying/ovulation order on offspring performance and lifespan. The results also suggest that ageing trajectories might be set up during very early stages of embryonic development.

In our study, egg size increased with laying order which support previous studies in this species^30^,^35^. The increase in egg mass with laying order may reflect a maternal strategy to counteract the negative effects of hatching asynchrony on offspring survival^36^. Similarly, the differences in size between male and female eggs suggest that females are also able to differentiate between the sexes of embryos, potentially investing more resources into the sex, in this case the males, with the higher energetic demands during embryonic development^37^, so compensating for their lower hatching success^36^.

The shortening of TL with laying order suggests that, even during the first stages of embryonic development, last-produced embryos within a clutch may experience a faster rate of cellular senescence^3^. This early with-clutch reduction in TL may explain the recently reported variation in early postnatal telomere dynamics between the first and last-hatched nestlings^38^. From a mechanistic point of view, such early variation in embryonic TL could be caused by different, but not mutually exclusive, mechanisms operating before and after fertilization. Those operating in the unfertilized follicle might affect the re-setting of TL that occurs at (or shortly after) fertilization. For example, previous studies have suggested that as a consequence of the ovulation process, the female ovaries become exposed to high levels of oxidative stress^39^,^40^; the duration of exposure to oxidative stress might therefore be longer in later ovulated oocytes/follicles, so causing a greater loss of TL prior to fertilization^2^. Since TL seems to be maternally inherited in birds^41^, it is possible that the later the ovulation of an oocyte/follicle, the shorter the TL inherited by the embryo. However, this is unlikely to fully explain our results since we observed a decline in telomere length within clutches but not across successive clutches produced by the same female, as would be expected under this scenario (indeed, TL increased from the first to the second clutch laid). It is also possible that the effect arises through the male germline: if a female ceases copulating upon commencing incubation then the sperm that fertilize her last-laid eggs may have been stored in her reproductive tract for longer, potentially exposing sperm cells to increased levels of oxidative stress^42^ and potentially shortening their TL^2^. Although nothing is known of the dynamics of TL in stored sperm, or the survival of sperm with different TL, this could be important in large clutches (>4 eggs) since female zebra finches usually lay an egg every 24 hr but usually cease copulation 72 hr after the first egg has been laid^43^; the sperm used to inseminate the last eggs in larger clutches is therefore likely to have been stored in the female reproductive track for several days (they can store sperm for approximately 2 weeks^43^). While we did not find a significant interaction between clutch size and laying order on embryonic TL, further studies are needed to investigate whether sperm storage has any influence on embryonic TL.

With regard to mechanisms operating after fertilization, it is possible that differences in telomere loss occurred in the first hours of embryonic development as a result of variation in egg composition. In birds such as the zebra finch important maternally-derived antioxidants such as vitamins E or A or carotenoids decline markedly with laying order^30^. These antioxidants play an important function in protecting the vulnerable lipid-rich tissues of bird embryos from the increasing levels of oxidative stress they encounter during prenatal development^21^,^44^. Consequently, it is likely that last-produced embryos are exposed to higher levels of oxidative stress during development, which in turn, could accelerate the post-fertilization loss of TL via several pathways such as the formation of 8-oxo-7,8-dihydro-2′-deoxyguanosine (8-oxodG) or through the generation of telomeric single and double-strand breaks (reviewed by^2^). In addition, later embryos within a clutch often have higher levels of maternal glucocorticoid hormones i.e. corticosterone^45^,^46^. These stress hormones may contribute to a reduction in TL in the developing embryos through increasing metabolism^47^, promoting a higher production of pro-oxidant damaging molecules^48^ or impairing DNA repair mechanisms^49^. Indeed, this is consistent with previous findings of Tissier *et al*.^50^ in the same species, where an increase in maternal corticosterone levels was associated with shorter postnatal TL in the offspring (see^13^ for similar effects on other bird species). Furthermore, the lower level of oestrogens in the later laid eggs of zebra finch clutches^51^ may have also reduced the transcription of genes encoding the enzyme telomerase, which restores TL, particularly during early stages of embryonic development. Maternal oestrogen levels during early gestation have been recently shown to predict TL in infants^52^. Our results suggest that TL can be influenced very early in development by maternal (and possibly paternal) effects that are independent of parental age, and that these could underlie poor subsequent performance and faster ageing rates. Given the lack of information regarding the factors influencing TL in embryos, we encourage future studies to investigate the isolated and synergic contribution of extra genomic egg substances such as maternal antioxidants and hormones on embryonic telomere dynamics.

The fact that TL in the embryos from the parents’ second clutches were, on average, longer that those from the first ones suggests that either increased breeding experience or an increase in parental age at conception may have a significant and positive effect on embryo TL. Indeed, previous work on birds and humans has shown that the mother’s age correlates positively with offspring TL^15^ and that increased paternal age can be associated with longer offspring TL, an effect thought to involve increased TL in the germline of older males^16^. Hence, it might be argued that the effect of clutch number on embryonic TL could be due to an increase in the parent’s age with clutch number. However, the time elapsed between the first and second clutches was only 4 weeks, which is perhaps too short for this to be a sufficient age difference given that zebra finches can live for over 8 years^9^. Alternatively, because all birds had no previous breeding experience at the start of the experiment, a more plausible explanation is a change in egg composition with breeding experience, since this is often associated with improved reproductive performance (e.g. better hatching success) even when parental age is controlled for^53^.

In conclusion, this study indicates that embryonic TL declines markedly with laying order within a clutch, to an extent likely to have a substantial effect on the offspring’s ageing and life history trajectories. Moreover, we provide evidence suggesting that this effect of laying order on TL is persistent so can have carryover effects on postnatal telomere dynamics. More longitudinal studies are needed to confirm whether parental effects and environmental conditions occurring during early stages of embryonic development permanently constraint telomere dynamics and presumably, impair individual lifespan.

## Methods

### Ethics statement

This study adheres to the animal welfare standards of the U.K. Home Office. All animal experiments were conducted in accordance with the Guiding Principles for the Care and Use of Laboratory Animals. The study was granted by Home Office Project Licence No. 60/4109 and approved by the University of Glasgow local ethical review committee.

### Animals housing and breeding conditions

Forty six adult male and female zebra finches of similar age (age range 482–519 days) were formed into 23 pairs. All of these parent birds were first-time breeders at the start of the experiment and were part of a previous study investigating the effect of early life dietary antioxidant availability on postnatal telomere dynamics (a detailed description of the origin of the birds and the dietary treatments is provided in the Supplementary Information and 12). Pairs were placed in individual breeding cages (60 × 50 × 50 cm) equipped with an external nest-box and coconut fibre as nesting material. Breeding pairs (n = 23) were maintained in standardized light conditions (16 L: 8 D cycle) and temperature (22.5 ± 2 °C), and provided with commercial seed mix (Johnson & Jeff, U.K.), oyster shell grit, cuttlefish and water. Once a week the birds also received Calcivet calcium supplement (Vetafarm, Wagga Wagga, NSW, Australia), a protein conditioning supplement (J.E. Haith, Cleethorpes, U.K.) and fresh vegetables. All pairs were formed on the same day and were of unfamiliar and genetically unrelated birds. Both male and female body mass was measured (±0.01 g) on the day they were paired.

Nest-boxes were inspected daily between 7:00–10:00 h and any new egg was marked and weighed using an electronic balance (±0.01 g). Zebra finches lay one egg each day, mostly within the first two hours after ‘dawn’ i.e. 06.00 am in our facilities^29^. Since zebra finches start incubation before the clutch is completed and therefore, embryos may differ in their development time and growth rate^54^, new laid eggs were switched for a dummy clay egg and immediately placed in an external incubator maintained at 37.5 °C and 80% Relative Humidity (Octagon 20 ECO Incubator; Brinsea Products Ltd, Standford). This allowed us to standardize the development conditions for all embryos as well as to remove any potential source of variation due to differences in the incubation behaviour among experimental pairs i.e. incubation temperature or length of the incubation bouts^55^. During avian embryogenesis, telomerase activity is strongly upregulated between the gastrula and neurula stages^56^, which are normally reached within the first 48 h of incubation in zebra finches^57^. Hence, all eggs were artificially incubated for 72 hr to allow enough time to all embryos to reach the neurula stage. Eggs were then removed from the incubator and stored at −80 °C for later molecular determination of embryo sex and embryo telomere length measurements (see below). Clutches were considered complete if no new eggs were laid for 4 days^58^. All breeding pairs were separated once the first clutch was completed and maintained in single-sex groups. Four weeks after this first reproductive event, the adult breeders were re-paired among them but ensuring that none of them shared the same partner as that they had during the previous reproductive event. As before, mates were both unrelated and unfamiliar and every freshly laid egg was removed, weighed ( ± 0.01 g), externally incubated for 72 h and then, stored at −80 °C. This second reproductive event allowed us to confirm whether or not the effect of laying order on embryonic TL continued across successive clutches, and to separate within-clutch effects from effects of parental age. Two pairs (one during the 1^st^ and another during the 2^nd^ breeding round) did not reproduce during the experiment.

To investigate the extent to which any effect of laying order on TL persisted into postnatal development and later life, we also examined the TL of red blood cells (RBCs) from a cohort of adult birds from the same captive population whose laying order within a clutch was known and which were each blood-sampled three times in early life (at 20, 40 and the last sample being at 90 days of age, when the birds were fully developed and sexually mature but had yet to breed). Details of the rearing regime for these birds are given in Noguera *et al*.^12^ and Supplementary Information.

### Telomere length analyses

DNA from the whole embryo or red blood cells (RBCs) was extracted by commercial kits (Macherey-Nagel, Bethlehem, PA, USA) in all the eggs that were fertilised (74 from the 1^st^ and 77 from the 2^nd^ breeding event). Whole embryo or RBC TL was then quantified by the qPCR method described in Criscuolo *et al*.^59^. The relative TL of each embryo sample was measured by determining the ratio (T/S) of telomere repeat copy number (T) to single control gene copy number (S), relative to a reference sample. Glyceraldehyde-3-phosphate dehydrogenase (GAPDH) was used as the single control gene. The telomere and GAPDH reactions were carried out on separate plates, and in both reactions the number of PCR cycles (Ct) required for the products to accumulate enough fluorescent signal to cross a threshold was determined. A detailed description of the PCR conditions is provided in^59^. In all cases, reactions’ efficiencies were within an acceptable range (GAPDH_Embryos_: 99.75 ± 3.77 SD, range: 95–104%; GAPDH_RBCs_: 101.92 ± 5.12 SD, range: 92–112%; TEL_Embryos_: 101.8 ± 3.45 SD, range: 97–106%; TEL_RBCs_: 106.71 ± 5.20 SD, range: 95–112%) and samples fell within the bounds of the standard curve (from 40 to 125 ng of DNA). All samples, including the standard curve, were run in triplicate and Ct values were used to calculate the relative T/S ratios, controlling for plate efficiency as describe in Pfaffl^60^ [Intra-class correlation coefficient (ICC); Embryonic TL: r = 0.84, p < 0.001, N = 151; RBCs TL: r = 0.87, p < 0.001, N = 357].

### Statistical analyses

Embryo data: We investigated the influence of within-clutch ovulation (laying) order on egg size (egg mass) and embryo TL using linear mixed effect models (LMM). The models included the clutch number (two levels; 1^st^ or 2^nd^ clutch of the mother) and the sex of the embryo as fixed factors, and the embryo’s position within the clutch (=laying order) and clutch size as covariates. Two-way interactions between fixed factors and covariates were also tested. Since mothers in better body condition (body mass) may allocate more resources into their eggs, we also controlled for this confounding effect in all models by including the mother´s body mass at day of pairing as a covariate. In the model examining effects on embryonic TL, egg size was also included as a covariate. In both models, the identities of the parents were included as a random factor to account for non-independence of eggs from the same mother and/or father.

Post-natal data: As these birds were part of a previous experiment (described in^12^), we ran a repeated measures mixed model to assess the effect of laying order on postnatal telomere dynamics (20, 40 and 90 days of age), while controlling for all previous factors that had previously found to have a significant influence on postnatal telomere dynamics (see^12^ and Supplementary Information for a detailed description of statistical results; Table S1). The model included birds’ identity as a subject term, age as a repeated measure factor and clutch size and laying order as covariates. All three and two-way interactions among age, clutch size and laying order were also tested. It should be noted that all the above models reflect exploratory rather than hypothesis-testing analyses.

Multicollinearity diagnostics were examined in all models by calculating collinearity tolerance values; these ranged from 0.79 to 0.97 indicating that the degrees of multicollinearity among the independent variables were acceptable. Residuals obtained from the models were always normally distributed. Analyses used Satterthwaite’s approximation for degrees of freedom and were simplified by removal of non-significant terms (in a backward deletion procedure), starting from three-way interactions; significance was estimated when terms were dropped from the model. Data are presented as means ± standard error, and the significance level was set at P = 0.05.

## Additional Information

**How to cite this article**: Noguera, J. C. *et al*. Embryonic and postnatal telomere length decrease with ovulation order within clutches. *Sci. Rep.* **6**, 25915; doi: 10.1038/srep25915 (2016).

## Supplementary Material

Supplementary Information

## Figures and Tables

**Table 1:** Summary of the final general linear mixed effect models (LMMs) of egg mass and embryonic TL.

Dependent variable	Source of variation	Parameter estimate	F	*df*_n,d_	p-value
Egg mass	Intercept	1.207			
	Laying order	0.012	10.052	1,123.02	0.002
	Sex (female)	−0.025	5.051	1,130.23	0.026
Prenatal (embryonic) TL	Intercept	1.272			
	Laying order	−0.050	10.893	1,137.75	0.001
	Clutch number (2^nd^)	0.123	7.336	1,141.08	0.008
