# Supplementary material for: Embryonic and postnatal telomere length decrease with ovulation order within clutches
Source: Sci Rep. 2016 May 13;6:25915. doi: 10.1038/srep25915 (PMC4865837; doi:10.1038/srep25915)
Supplement: Supplementary Information [file srep25915-s1.pdf]

**Noguera et al. '**EMBRYONIC AND POSTNATAL TELOMERE LENGTH DECREASE WITH OVULATION ORDER WITHIN CLUTCHES'

José C. Noguera , Neil B. Metcalfe, Sophie Reichert, Pat Monaghan

# SUPPLEMENTARY INFORMATION

*Origin and previous experience of the adult breeders*

The adult breeders used to produce experimental clutches were domesticated zebra finches that were part of a previous study investigating the effect of the availability of dietary antioxidants on postnatal telomere dynamics (see [1] for a full description of the dietary treatments). In that study 36 zebra finch broods were randomly assigned to a 'High' (H) or 'Low' (L) antioxidant dietary treatment until the birds were 40 days old (growth period). At 40 days of age, half of each brood was assigned to follow the same (oral) antioxidant treatment as during the first 40 days of life, and half to the opposite treatment from day 41 onwards (sexual maturation period). At 90 days, when birds were adult and sexually mature, the dietary treatment ceased and all birds continued being fed with a standard aviary diet. All birds were blood sampled at 20, 40 and 90 days of age, and their telomere length measured by qPCR (a full detailed description of telomere analyses is provided in [1] and the main text). Only the birds that were reared on the same antioxidant diet (L or H; 23 males and 23 females) from day 1 to day 90 [hereafter referred as 'Low' (L) or 'High' (H), respectively] were used during the reproductive trials to produce experimental clutches (see main text).

*Breeding scheme*

The first clutch was produced by pairing half of the males in each dietary treatment group (L or H) with a female of the same dietary treatment as themselves, and the other half with a female of the opposite treatment; all possible combinations of parental dietary treatments were equally represented. Both male and female body mass was measured (± 0.01 g) on the day they were paired. All pairs were formed on the same day and were between unfamiliar and genetically unrelated birds. All breeding cages were equipped with an external nest-box and coconut fibre as nesting material. Commercial seed mix and water were provided *ad libitum*. Nest-boxes were inspected once daily between 7:00-10:00 h and any new egg was marked, switched for a dummy clay egg, weighed using an electronic balance (±0.01 g) and transferred to the external incubator (see main text). All pairs were separated once the first clutch was completed (if no new eggs were laid for 4 days [2]). Approximately 4 weeks after this first reproductive event, males were re-paired and allowed to produce a clutch for a second time, but this time with a new female of the opposite dietary treatment to the one they had previously. As before, mates were both unrelated and unfamiliar. The age at which the birds were paired in the two breeding events did not differ between experimental groups or sexes [First reproductive event (LMM): treatment, $F_{1,17.32}=0.21$, $p=0.647$; sex, $F_{1,16.51}=0.07$, $p=0.791$; treatment x sex, $F_{1,16.86}=0.56$, $p=0.464$; identity of the family of origin (random factor), $Z=3.63$, $p<0.001$; Second reproductive event (LMM): treatment, $F_{1,17.36}=0.34$, $p=0.565$; sex, $F_{1,16.56}=0.10$, $p=0.739$; treatment x sex, $F_{1,16.05}=0.23$, $p=0.63$; family of origin, $Z=3.63$, $p<0.001$]. The same was true of body mass on the day of pairing [First reproductive event (LMM): treatment, $F_{1,32.17}=0.151$, $p=0.700$; sex, $F_{1,37.43}=1.35$, $p=0.251$; treatment x sex, $F_{1,34.34}=0.438$, $p=0.513$; identity of the family of origin, $Z=0.421$, $p=0.674$; Second reproductive event (LMM): treatment, $F_{1,36.92}=0.635$, $p=0.431$; sex,

$F_{1,38.28}$=2.816, p=0.101; treatment x sex, $F_{1,41.34}$=0.01, p=0.930; identity of the family of origin, Z=0.954, p=0.340].

*Effect of parental dietary treatment on embryo telomere length*

In order to check that the results of the models testing the effect of laying order on egg mass and embryo telomere length were not influenced by the earlier dietary manipulation received by the adult breeder, we re-ran the original models (see main text) but including the maternal and paternal dietary treatment (L or H) as well as their 2-way interaction as fixed factors. The analyses gave the same results as the original models reported in the main text, as neither the paternal nor maternal diet or their two-way interaction had significant effects on egg mass (paternal diet: $F_{1,14.37}$=2.393, p=0.144; maternal diet: $F_{1,16.12}$=0.229, p=0.639; paternal x maternal diet: $F_{1,85.40}$=2.098, p=0.151), or embryo telomere length (paternal diet: $F_{1,13.14}$=1.218, p=0.289; maternal diet: $F_{1,15.25}$=0.001, p=0.979; paternal x maternal diet: $F_{1,122.83}$=0.602, p=0.439).

*Testing the effect of laying order on postnatal telomere dynamics*

To investigate the extent to which any effect of laying order on embryo TL persisted into postnatal development and later life, we examined the existing longitudinal dataset of TL in red blood cells (RBCs) of the birds used by Noguera et al.[1]; note that the effect of laying order was not examined in that previous analysis. As described above, the birds were part of an experiment where the effect of nutritional conditions during development on TL at 20,

40 and 90 days of age was investigated. With this database, we ran a repeated measures mixed model with the birds' identity as a subject term, age as a repeated measure factor and laying order and clutch size as covariates. Three and two-way interactions among age, laying order and clutch size were also tested. In the model, we also controlled for all previous factors that had previously found to have a significant influence on postnatal telomere length (see [1]), i.e. dietary treatments (during the growth and sexual maturation period), sex, body mass and the interaction between sex and dietary treatment during sexual maturation (see [1]). The model was fit with Satterthwaite's approximation for degrees of freedom and simplified by removal of non-significant terms (in a backward deletion procedure), starting from three-way interactions.

The summary of the repeated measures mixed model is shown in Table S1. Postnatal telomere length shortened with age (Table S1) but importantly, after controlling for all factors that had a significant influence on postnatal telomere length, there was still a strong effect of laying order on telomere length (see Table S1). Thus, postnatal telomere length decreased with laying order (Table S1), and the effect was persistent across all ages (laying order x age: $F_{2,248.32}$=0.203, p=0.816). Neither clutch size ($F_{1,321.62}$=1.323, p=0.251), body mass ($F_{1,243.68}$=1.773, p=0.184) nor any three and two-way interactions among age, laying order and clutch size were statistically significant (all p>0.367).

Table S1. Summary of the final repeated measures mixed model of postnatal telomere length. Note that 'treatment (sexual maturation)' refers to the dietary treatment that the birds received during the period of sexual maturation (40-90 days).

| Dependent variable | Source of variation | Parameter estimate | F | $df_{n,d}$ | p-value |
|---|---|---|---|---|---|
| Telomere length (RBCs) | Intercept | 0.986 | | | |
| | Age (20 days) | 0.326 | 41.911 | 2,254.10 | <0.001 |
| | (40 days) | 0.070 | | | |
| | Laying order | -0.044 | 18.726 | 1,326.61 | **<0.001** |
| | Sex (female) | 0.165 | 2.568 | 1,324.92 | 0.110 |
| | Treatment (sexual maturation) (L) | 0.037 | 6.791 | 1,325.05 | 0.010 |
| | Sex x treatment (sexual maturation) | -0.233 | 14.987 | 1,324.54 | <0.001 |

Full model: age + laying order+ clutch size + body mass + sex + treatment (growth period) + treatment (sexual maturation) + age x laying order + age x clutch size + sex x treatment (sexual maturation) + age x laying order x clutch size.

**Figure S1**. **Relationship between the position of a zebra finch egg within a clutch (i.e. its laying order) and its mass and TL after 72h of incubation (N=151).** (a) Egg mass (mean ± SE) increased with laying order whereas (b) TL in early-stage embryos (mean ± SE) decreased with position of the egg in the laying order within the clutch, irrespective of clutch size. Solid lines show linear regressions and data points are presented as circles; see text for statistical analysis.

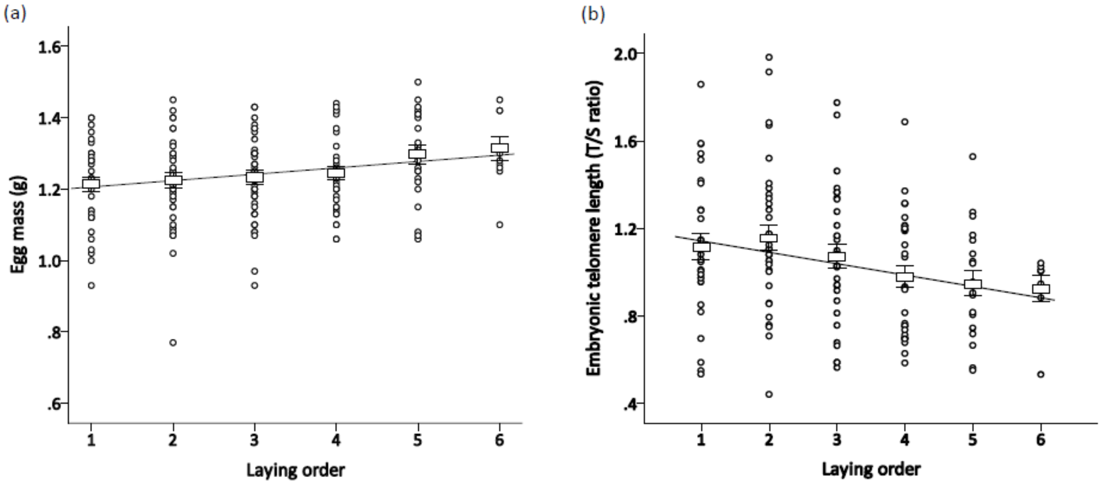


**Figure S2**. **Early-stage embryo TL in successive clutches (N=151)**. On average, embryos from the first clutches of female zebra finches had shorter TL than those from the second clutches. Boxes represent mean ± SE and data points are presented as circles.

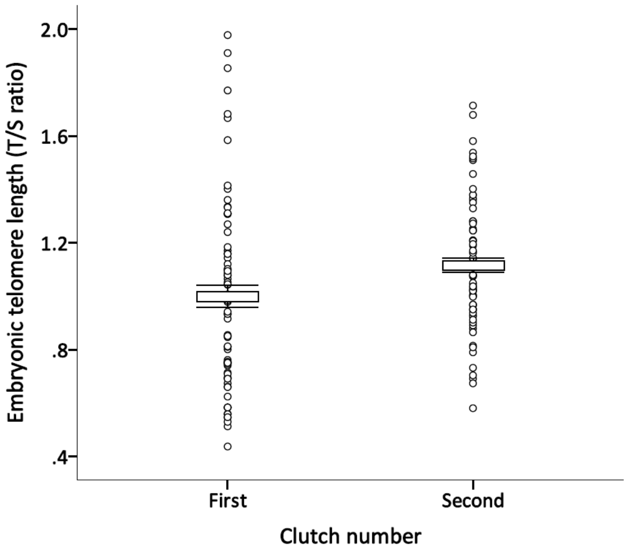

**Figure S3**. **Laying order and postnatal telomere dynamics (N=122).** Relationship between the original position of a zebra finch within a clutch (i.e. its laying order) and its telomere length (mean ± SE) at 20 (white box, dashed line and white circles), 40 (grey box, solid grey line and grey circles) and 90 days of age (black box, solid black line and black circles). Lines show the adjusted regression lines and data points are presented as circles. There was a significant effect of laying order on telomere length which was not affected by age (see text for statistical analysis).

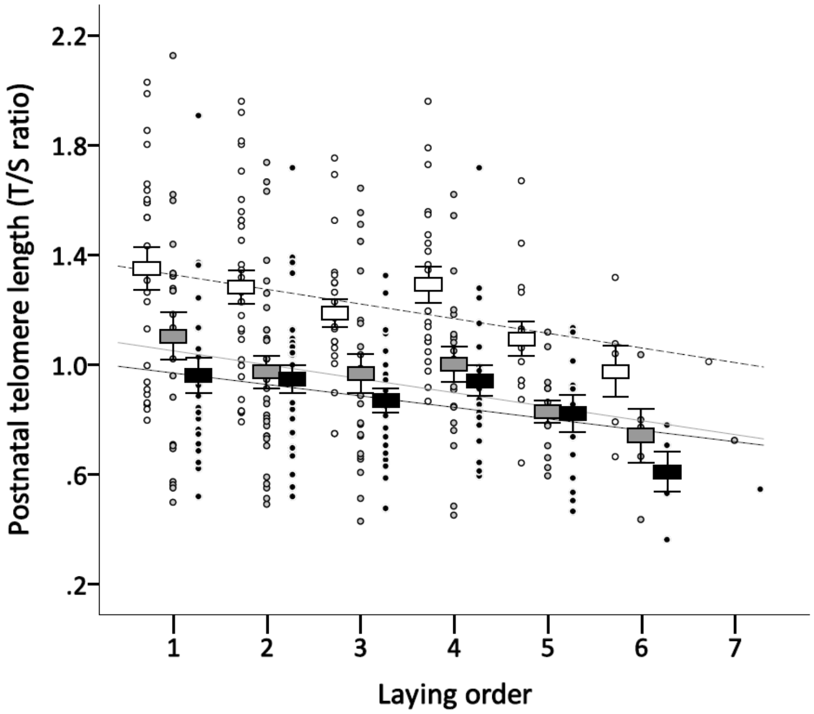

## REFERENCES

1 Noguera, J. C., Metcalfe, N. B., Boner, W. & Monaghan, P. Sex-dependent effects of nutrition on telomere dynamics in zebra finches (*Taeniopygia guttata*). *Biol. Lett.* **11**, 20140938 (2015).

2 Blount, J. D., Metcalfe, N. B., Arnold, K. E., Surai, P. F. & Monaghan, P. Effects of neonatal nutrition on adult reproduction in a passerine bird. *Ibis* **148**, 509-514 (2006).
